# Extracts and Gleanings

**Published:** 1872-07

**Authors:** 


### PART II.

### Abridged Extracts and Gleanings fro mour Exchanges

### MULTUM IN PARVO.

Bromide of Potash for Hysteric Gastralgia Consequent Upon Change of Life.—In an interesting case of the peculiar pain, of which women so often complain when passing the change of life, which they often name as colic, I gave immediate and, I think, permanent relief, by the use of Bromide of Potash. This first dose (consisting of thirty grains) was given after the attack had commenced—it acted like a charm—the after practice consisted in taking a dose of Bromide at nightfall (as the paroxysms usually came on after night.) The lady has never felt a return of pain since; she now takes only an occasional dose. For four years, I had tried in turn, opiates, mercurials, emetics and cathartics, without avail. Geo. F. Taylor.

Sulphurous Acid Lotion in the Treatment of Contused Wounds.—Dr. John Balfour states that an extended experience has given him great faith in this application. It gives almost instant relief from pain, controls and greatly restrains suppurative action, and, where possible, secures primary union perhaps as efficiently as carbolic acid. The lotion is of the strength of one in twelve; a thin rag (the thinner the better) should be laid over the wound, and kept constantly wet for the first thirty-six to forty-eight hours. When cold becomes less agreeable, the lotion is used tepid, the rag being wetted every twelve hours and covered with gutta percha. Where primary union is taking place, about the third or fourth day, a dressing of zinc ointment is to be substituted for the washing: this allows the skin to heal. When suppuration is established, a zinc lotion may be used after a week or ten days, and the cure wrought out on ordinary principles. Dr. Balfour records the following, amongst other cases :—S. B., a lad between eleven and twelve years of age, on the 8th of June, in company with some other boys, was amusing himself with gunpowder; a "Peeoge" (or devil) hung fire, and he poured some powder on it from the flask. This of course exploded, and tore open the metacarpal space between the thumb and forefinger of the right hand. The metacarpal bone of the thumb was fractured, and both wrists scorched. A mass of the short flexors of the thumb was forced out of the wound, contused, torn and blackened. As this muscular substance was much injured and could not be returned without using undue force, a good deal of it was cut off; the wound was washed out with the sulphurous acid lotion, covered with a rag wet with the same, and the fracture was kept in position by tying the thumb to the forefinger. Had a fair night's rest; the wrists (not complained of yesterday) now painful and beginning to vesicate; dressed with carbolic acid and oil. Everything went on well, and the burns on the wrists healed kindly, suppuration was most moderate, cicatrization rapid and perfect. Dr. Balfour lately passed the boy into a public work, with a thumb very little, if it all, the worse for the accident.—*Edinburgh Medical and Surgical Journal*, *Nov.* '71

FOR GASTRALGIA.—Prof. C. C. Cox recommends the following:

R.—Tinct. Opii,
Tinct. Capsici,
Æther Sulphi,
Tinct. Camphori, . . . . aa dr. iij,
Chloroformi, . . . . . dr. j.—M.
S. Teaspoonful, pre re nata.

The efficacy of the above is much increased if taken in a wine-glass of hot ginger tea.

STIMULATING HYPODERMIC INJECTIONS IN TYPHOID FEVER.—We find in the *Gazette Hebdomadaire* of June 16th, some account of a novel mode of treatment adopted by a German physician in the collapse of typhoid fever. The subjects were Prussian soldiers, who, during the siege of Paris, suffered from typhoid of extremely ataxic type, with feeble heart-movement, small and irregular pulse, cyanosis, cold extremeties, and general collapse. Dr. Zuelzer, observing the resemblance to the choleric condition, in which he had derived good results from stimulating hypodermic injections, determined to try the same method, and accordingly injected into each of the four extremities ten drops sulphuric ether, with five drops "anisated ammoniacal solution." "The results were remarkable. The pulse, before small and irregular, became full and strong; the contractions of the heart, which had been feeble and irregular, became energetic and regular; its impulse, before imperceptible, was now well marked. Frequently, after one or two injections, the cyanosis and collapse vanished. This plan has the additional advantage of gaining time for other treatment. Small abscesses are formed at the places of puncture, but they are of no importance."

FOR LEUCHORRHŒA.—

| | | |
|---|---|---|
| ℞.—Extracti Hyosciami, | . . . | dr. j. |
| Argenti Nitratis, | . . . . | gr. x. |
| Cantharidis pulv., | . . . . | gr. xij. |
| Quiniæ Disulphatis, | . . . | scru. ij.—M. |
| ft. pil. no. 40. | | |

S. One morning and night.

These are highly recommended by an intelligent and experienced practitioner in the treatment of leuchorrhœa, occurring in anemic and nervous females.

PESSARY FOR OFFENSIVE UTERINE DISCHARGES.—We understand that Dr. John Day, of Geelong, has recently been using with great advantage a new kind of pessary as a means of removing the offensive odour given off in certain uterine diseases, as cancer, etc. These pessaries are made by gently melting cocoa butter, and then adding to it ozonic ether in the proportion of a drachm of the latter to an ounce of the former. The addition of about one-eighth part of white wax will give greater solidity to the mass, and in hut weather seems an improvement. Each pessary should weigh about a drachm and a half. The mass thus prepared retains its active agent, the peroxide of hydrogen, for a month or longer, as may be readily proved by scraping a few fine shaving from a pessary on a drop of blood or pus lying on a piece of white paper, then folding the paper, and holding it between the forefinger and thumb for a minute or two, so as to dissolve the cocoa butter, and lastly adding a drop of tincture of guaiacum (oxidized if pus is used), when a blue reaction will at once take place.—*British Med. Journ.*, Oct. 28, 1871.

Constant Galvanic Current in Treatment of Neuralgia.—1. The first case occurred to a well-known merchant in Manchester. He was forty years old, and came to me on the 15th of June last, with intense frontal neuralgia, which had lasted a fortnight. It was most severe in the supraorbital branches of frontal nerve. Five minutes' gentle application of the continuous current by means of small wet sponges attached to small conical conductors sufficed to cure him of his torment on the spot, and it never returned. "I wish," he said the next day to me, "I had known you a fortnight ago. I should have had a very different Whitweek to what I have had!"

II. The second case I shall mention occurred in a youug lady, a governess with a leading merchant's family in this neighborhood. She had intense neuralgia of the left side of the face, which she attributed to the partial extraction of a tooth ten days before. I applied the current as in the last case, but with slender hopes of relieving her. One application, however, completely freed her from pain for a fortnight; and then she returned with it again, when a similar application effectually removed it, and there has been no return.

III. My third case occurred on the 4th of this present October, to a valued female servant of an old patient of mine. She was a girl twenty-four years of age, and had been suffering from facial neuralgia of both sides for three months. For two nights previously to coming to me, she had been compelled to get up from bed and come down stairs and spend the night awake, and in great agony. Five minutes' application cured one side of the face, and a second five minutes' application cured the other, and then, to my surprise, "tired Nature's sweet restorer" overwhelmed the poor girl, and she fell fast asleep in her chair at my house. And here, again, there has been not the slightest attempt at return of pain.

These three cases have been selected from the records of my practice in the use of electrics during the last two years in the cure of disease to illustrate the truly marvelous power which the constant galvanic current has over pain. The battery used in all three was Weiss's, sometimes known as Foveaux's. I used about eight cells of this splendid battery, with very small sponges (about as large as would fill the end of a thimble) soaked in warm water, and fixed, as I have just said, to those small conical electrodes which are used for the localization of the current in paralysis of the interossei and lumbricales muscles. I applied them to the part where the pain was, keeping them about one inch apart from each other. I moved them about, but did not *remove* them, or either of them, from the skin for about two minutes. I then rested for one minute, and applied them again for two minutes more. If the pain had not been removed I should have gone on for another five minutes. But as soon as the pain ceases in a case of neuralgia I make it a rule immediately to discontinue the application. In each of these cases I directed the patient to come daily for a repetition of the operation. But, happily, there was no need for this.—*Joseph Stead, M. R. C. S. E., Medical Press and Circular.*

SLEEPLESSNESS occurring in hypochondria, hysteria, and indeed, in all nervous affections, may be overcome with great certainty by the administration of the following pills:

R.—Assafœtidæ, . . . . . dr. j.

Morph. Sulphatis, . . . . . gr. ij.—M.

ft. pil. no. 30.

S. One or two at bed time.

They are very effecacious in arresting the dry cough which is occasionally consequent on disordered menstruation in nervous females.

CHLOROFORM IN LABOR.—Dr. Graily Hewitt, in the Obstetrical Society of London (*Medical Press and Circular*) uttered the following language: "We have come (some of us, at all events,) to recognize the fact that chloroform has a tendency to make labor *lingering;* that it sometimes enfeebles the uterus, and may thus cause hemorrhage. This tendency it is proposed to do away with, by diluting the chloroform by mixture of alcohol or other vapors, or by accurate mixture with air. Dr. Sansom has pointed out the great liability to the inhalation of poisonously high percentages of chloroform at high temperatures, unless proper care be exercised. Mr. Ellis has given us new inhalers for effecting such mixtures. The general conclusion I take to be, that in ordinary midwifery practice, the anæsthetic should be diluted; that it should not be given to produce the full effect, and that in all cases, rather excessive precautions against hemorrhage are required when chloroform is given.—*Virginia Clinical Record.*

CHLORAL IN URINO-GENITARY DISEASES.—In *incontinence of urine*, Dr. W. Thomson has recorded two cases of long duration which were *rapidly* cured by chloral (*Lancet*, Nov. 19, 1870,) and Dr. Bradbury has met with similar success. Dr. Bradbury also recommends it, and has been successful in the treatment of nocturnal *incontinence of semen.* In both these affections he considers chloral to possess marked advantages over balladonna.—*Med. Surg. Journal.*

Preservation of Tinct. Kino from Gelatinizing.—Dr. J. W. Wood, in the *American Journal of Pharmacy*, says:

Among all our tinctures perhaps there is not one so liable to deteriorate by exposure or by long keeping as tincture of kino, made in accordance with the U. S. Pharmacopœia. Its well known property of gelatinizing in a short time—a property which yet remains to be investigated, it being thereby rendered inert—precludes it from being as extensively used as its virtues would seem to warrant. This property renders it inadmissible when we desire a reliable tincture to prepare it in large quantities.

The pharmacopœia formerly directed it to be prepared with dilute alcohol as the menstruum; but later it was thought to be of advantage to increase the proportion of alcohol to two-thirds, yet it is doubtful if there was much gained by this change.

I would therefore submit the following mode of preparation, which I consider, from the experience I have had, will meet with the desired end, and up to the present time results do not seem to disprove it. It is as follows:—

| | |
|---|---|
| R.—Kino, in fine powder, . . . | oz. 1½. |
| Alcohol, 835, fl. . . . . | oz. 8. |
| Aquæ, fl. . . . . . | oz. 4. |
| Glycerinæ, fl. . . . . . | oz. 4. |

Mix the alcohol, water and glycerine together, and having mixed the kino with an equal bulk of clean sand, place the mixture in a percolator and pour on the menstruum.

This menstruum seems to thoroughly exhaust the drug of its astringent principle, and also makes a nice-looking preparation.

Some which I made on the sixteenth day of July, 1870, was exposed to the influence of the atmosphere, the stopper of the bottle containing it having been removed for several months, so that it had evaporated to at least two-thirds; yet it remains as good as when freshly made, without any apparent tendency to gelatinize.

The menstruum might be somewhat modified, perhaps, with advantage; as, for instance, by using proportionally less alcohol and more glycerine and water, or *vice versa.* At any rate, I will give it for what it is worth, adding at the same time the suggestion—and it is only a suggestion—that the same menstruum be employed in preparing tinct. catechu, which, though not so liaole to gelatinize as tinct. kino, yet sometimes does so.

Belladona Locally Applied in Orchitis and Other Inflammations.—Dr. S. D. Turney, in the *American Practitioner*, states that he has for sixteen years used belladonna as a local application in orchitis, and that in twenty-four hours there is usually entire relief, and the patient may leave his room, after having applied a suspensory bandage. The same remedy is also very efficacious in inflamed breasts. The formula is as follows:

℞.—Extract of belladonna, . . . . dr. 1.
Glycerole of starch, . . . . . oz. 1.

Mix and rub thoroughly over the scrotum and along the affected cord every three or four hours.

Local Application of the Leaves of Datura Stramonium.—In the *Revue do Therapeut. Medico-Chirurg.* for Nov. 15, 1871, Felix Neucourt praises cataplasms made of the leaves of Datura Stramonium as of great service in various inflammations, detailing a number of cases in which he found them very useful. These cases comprised acute intestinal inflammation, acute and chronic phlebitis, phlegmonous, muscular rheumatism, arthritis of the great toe, and inflammation of the vulva. The cataplasm should be applied immediately over the affected part, and be changed about three times a day.

In a case of cardiac dropsy the application of a cataplasm of stramonium to the abdomen produced, after eight hours, diarrhœa and profuse diuresis, with subsequent relief to the anasarca. In inflammatory cases the relief was most marked when the cataplasms were applied early. In no case were symptoms of mydriasis or narcotic intoxication induced.

For Menorrhagia.—Prof. Barker recommends the following:

℞.—Acidi gallici,
Acaciæ, pulv. . . . . . aa dr. ij.
Syrupi Tolutan,
Aquæ font. . . . . . aa f. oz. ij.—M.
S. Teaspoonful, a dose.

Chorea.—In a paper on chorea read before Medical Library and Journal Association of New York, (Medical Record) Dr. J. Lewis Smith, in referring to the treatment of this disorder, says:

Of the milder nervines the one which has the highest reputation among the French physicians is *valerian;* and, from the evidence adduced in its favor, there can be no doubt that it exerts a very beneficial influence upon the milder forms of the disease. *Assafœtida* also deserves attention.

In the severest and most obstinate cases, where there are not only congestion but also dilation of vessels, emboli, thrombi, and softening, the mineral astringents, especially the *oxide* and *sulphate of zinc*, may be employed. Dr. Hammond thinks that zinc is probably more used in this country than any other single remedy; he has himself employed it with good results in gradually increasing doses, from two or three grains up to twenty or thirty, three times a day. But the sulphate is so acrid and disagreeable that it should be used only in the above-mentioned cases.

It is rudely estimated that the proportion of cases of cerebral chorea varies from one-fourth to one third of the whole. The number of the rheumatic cases is nearly equal. The diagnosis of these latter is not very difficult, although it requires some nicety. It is well to remember that those cases in which rheumatism is rather latent than manifest are most apt to be attended with heart disease; that anæmic heart-murmurs should not be mistaken for disease of the valves; that chorea of the papillary muscles may produce such temporary changes in the shape of the auriculo-ventricular openings that a transient regurgitant murmur may be heard; that some pains and swellings of the joints may be caused by the muscular exertion alone; and that the urine may become so saturated and dense from the wear and tear of the muscular and nervous systems, as to resemble the urine of rheumatism. With all these precautions, some cases of rheumatic valvular disease will escape notice, for the auricular surfaces of the valves only may be roughened and no murmur may be produced.

In the rheumatic cases single doses of calomel may be used as purgatives when the bowels are constipated and the evacuations light in color. Subsequently Rochell salt may be relied upon. The *alkalies* and *aconite* and *colchicum* are also good remedies; but *actea racemosa* (cimicifuga) is our main reliance. This remedy, as stated by Dr. Smith, is mainly depended upon in Philadelphia, and usually it is tried in all cases indiscriminately. It is employed in large doses up to half a pint of the decoction (oz i. ad aq. Oi.) per day.

The *spinal* cases may often be detected by the tenderness over the spine, and for these the *ether spray* has been decreed to be the best remedy. Dr. Hammond says his success has been unequivocal when the atomized ether has been thrown upon the whole spine, from the occiput to the sacrum, for ten minutes every day —a cure being generally affected in two weeks.

The *reflex* cases come from various sources, the most numerous being due to the irritation of second dentition, worms, constipation, difficult establishment of menstruation, pregnancy, sexual irritation, and Onanism. The *bromide of potassium* for dental and sexual irritation is the best remedy. *Vermifuges*, such as santonin, turpentine, injections of quassia, for ascarides, etc., will suggest themselves to every mind.

In that very large class of cases which arise from a peculiar irritable debility of the great motor tract, from the corpora striata, through the spinal cord, to the very terminations of the motor nerves in the muscles, *strychnine* is the most important remedy. It is one of the best tonics to the motor centres, nerves, and muscles, and exerts an alterative action on many nervous and muscular affections: still it is decidedly wrong to use it empirically in all cases of chorea.

In the anæmic cases the preparations of *iron* are, of course, the most suitable medicines. They may be combined with conium in some instances where the muscular action is very great, and with aloes when there is constipation or amenorrhœa. But it is unscientific to rely upon iron in all cases, as has been done by Elliotson and others.

*Arsenic* is a nervous tonic, acting, according to Handfield Jones, on both the cerebro spinal and the organic system, increasing their functional power, and preventing congestion by contracting the vessels, especially those of the skin and uterus; similarly it is found to reduce an undue supply of blood to the spleen and bronchi, and it may act in the same way upon the vessels of the nervous centres. It may cure chorea and asthma by acting as a tonic of nerves of nervous centres which are weak and unduly excitable. Hence we need not wonder that arsenic enjoys a high reputation in the treatment of chorea; but, like the other remedies, it must not be regarded as a general specific for all cases.

Finally, in a small number of cases there is pain the head, with signs of cerebral congestion, and even of *meningitis*. It is to this class only that tartar-emetic, so highly lauded by West, is applicable.

Proper diet, cod-liver oil, and the phosphates, the tepid or cool plunge or shower-bath, are almost necessary adjuncts.

FOR DIARRHŒA.—

R.—Tinct. camphoræ, . . . f. oz iss.
Tinct. capsici, . . . . f. oz. ss.
Spiriti Lavendular, com.,
Tinct. opii., . . aa f. oz. j.—M.
S. 20 to 30 drops pro re nata.

This is highly recommended both in the milder forms of diarrhœa and in the early diarrhœal stage of epidemic cholera, by Dr. Horace Green, of New York.

The Use of Iron in Scarletina.—Dr. Russell Aldridg (British Medical Journal, August 12,) draws the attention of the profession to the use of iron in scarlatina. He has given it for the past two years with great success; so much so as to induce him to believe that in it we have a powerful remedial agent for that disease. He has found, if it be given as soon as the disease makes its appearance, that not only does it shorten and lessen the severity of the attack, but it also fortifies the patient against the after-consequences—dropsy, &c. The form which he has mostly used has been the liquor of pernitrate of iron, in syrup or glycerine, in doses of ten minims every three hours for children of from one to six years, increasing, according to age, to fifteen, twenty-five, or thirty minims. During convalescence, he has given citrate of iron and quinine, ammonio-citrate of iron, or syrup of phosphate of iron, according to circumstances. This, with the exception of warm fomentations to the neck in cases of scarlatina anginosa, is all the treatment he has adopted.—*Nashville Medical Journal.*

Diaphoretic and Expectorant Mixture.—The following was much used by the late Prof. Sam'l H. Dickson, in pneumonia, croup, bronchitis, etc.:

R.—Ipecac. pulv. . . . . . dr. j.
Infus. Rad. serpentaria, . . oz. vj.
Tinct. opii camph., . . . f. dr. ij.—M.
S. Teaspoonful, pro re nata.

Inunction of Newly-Born Infants.—At a recent meeting of the Lynn Medical Society, Mass., Dr. Nye spoke of the management of the child after birth, and reported one case which died of too long exposure in being washed, and another that was chilled and livid, but was saved by the hot bath. He recommends that the child be covered with lard and wiped, no water being used. In two recent cases we have substituted inunction for washing of the infants, not only at the time of birth, but long subsequently with manifest advantage. Fresh lard was used, and seemed to both protect and nourish the children, as they were very healthy and grew finely.—*The Medical Cosmos.*

Intestinal Obstruction from Accumulation of Fæces Cured by the Internal and External Use of Ice.—M. Prunac reports (L'Abeille Medicale, December 25; from Le Gazette des Hopitaux) a case in which urgent symptoms of intestinal obstruction, dependent upon this cause, yielded to the application of ice to the abdomen, and to injections of ice-water every quarter of an hour, after having resisted the more usual methods of treatment.

Hyperæsthesia of the Throat.—In a case of this malady Dr. C. Handfield Jones ordered twenty grains of muriate of ammonia with ten minims of mixture of belladonna in water every four hours, hot fomentations to the neck, and aconite liniment. She also had immediately fifteen minims of chlorodyne, which gave her two hours' sleep; and at 9 P. M. he found her decidedly better, though she had a good deal of strange pain, hardly describable, on both sides of her head. The dose of chlorodyne was repeated at 10 P. M., and in the course of the night she had fifteen grains of chloral; but she was in high fever and delirium all night long. The room seemed whirling round; she felt as if she must get up and go out—as if she should fly. She saw numbers of persons in the room, with great heads and little bodies. At 9.30 A. M. on the 10th, when he saw her, her throat was much better; she took some toast. The catamenia had reappeared, which had stopped, after a day of normal continuance, on September 3d. He ordered a drachm of tincture of cinchona flava four times a day. On the 11th the throat was much better; she had still dysæsthesiæ about the head, and a large patch of urticarious erythema had appeared about each elbow. On the 12th she was so well as to call on me and take her leave before setting out for the North. She had sweated copiously the preceding night, had a good deal of eruption on the legs and thighs, but made no complaint of her throat. The attendant neuralgia, the peculiar distress, the nocturnal delirium, the urticaria, and the "Juvantia," all go to prove the neurotic character of the disorder, and that it was no common sore throat, but one where the hyperæsthetic greatly predominated over the imflammatory element. The abrupt cessation of the catamenial flow may have had to do with the supervention of the disorder; but I think the most influential factor was influenza.

Emmenogogue.—

| | |
|---|---|
| R.—Ferri Sulphatis, . . . . | dr. iss. |
| Potaso Iodid, . . . . . . | dr. ij. |
| Tinct. Cardamon, | |
| Syr. Simp., . . . . . . | aa f. oz j. |
| Aquæ font., . . . . . | f. oz ij.—M. |

S.—Tablespoonful three times a day.

Of this Horace Green remarks: "In cases of suspended menstruation, attended with headache, and with pains in the pelvic regions, the above preparation may be advantageously administered."

Ipecac Enemata in Dysentery.—Dr. H. V. Ferrell (Clinic) says:

In July and August, 1870, during an epidemic of that disease in this section I treated thirteen cases of acute dysentery; the first and second in the usual manner. The first recovered, the second, a child, æt 18 mo., died. The third and fourth, parents of the child (their other child had already died of the disease under the common treatment by another physician), were treated with lead and opium till I thought they too would die, when I decided to use ipecac. It was administered as follows:

R.—Pulv. Ipecac. . . . . dr. i.
Mucilag. Acaciæ, . . . . oz. vi.—M.

Give as enema.

This was repeated three times a day for three days, when a complete cure was effected. In another case, male æt 40, its benefits were even more strikingly displayed.

Of the thirteen cases, two were treated without ipecac; one recovered and one died; three were evidently saved by it after the most approved treatment had entirely failed. In the remaining eight cases it was used early and alone, and in none did any symptoms arise betokening great danger.

In July last I treated Mrs. I., widow, æt about 50, for sporadic dysentery. Ipecac was administered per enema, and she made a hasty recovery—in her case, to relieve the excessive tormina, gtt. xv. fl. ext. hyosciami were added and had the desired effect.

Dr. Jessup gives 10 gr. of the powder with dr. ss. tr. opii in oz. ii. arrow root decoc. or mucilage three times a day. I have never given to an adult less than 60 gr. of the powder and in oz. vi. mucilage acacia or slippery elm which is as good, and can often be obtained when others cannot.

For General Anasarca Attended with Debility.—

R.—Juniperi fructi, . . . oz. ij.
Potass. nitrat., . . . . oz. ss.
Vini albi, . . . . O ij.—M.
Macerate 20 hours.

S. Two tablespoonfuls twice a day.

Quinine in Pneumonia.—Dr. Terrell, of Henrico county Virginia, is in the habit of treating pneumonia with large doses of quinine, and says that he has seen as much benefit (in a curative way) from this plan of treatment as from any medicine in any disease. It shortens the attack, and the earlier it is resorted to the better. He regards quinine as a great equalizer of the circulation, and, acting upon the nerves which control the circulation, it is necessarily anti-congestive. Some physicians, he remarks, admit its beneficial effects in typhoid pneumonia, or when there is a remittent tendency, or malarial complication in any form; but he makes no discrimination, giving it in *all* cases, in large doses, for its sedative and anti-congestive powers. For the diarrhœa which sometimes supervenes in the progress of an attack of pneumonia, he has found nothing better than a few grains of tannin, in combination with the quinine for arresting it.—*Med. Record.*

PILL FOR DYSMENORRHŒA.—

| | |
|---|---|
| R.—Extracti Belladonnæ, . . . | gr. viij. |
| Camphori pulv. . . . . . | dr. j. |
| Quiniæ Disulphatis, . . . . . | scr. ij.—M. |

Ft. pil. No. 30.

These pills are very effective in the treatment of dysmenorrhœa. One pill may be exhibited every hour or two till the pain ceases. In females of a nervous temperament, when painful menstruation occurs, independent of organic lesions, these pills administered as above directed, seldom fail to afford relief. In those cases, where a tonic is indicated, the following are more appropriate, and are equally efficaceous:

| | |
|---|---|
| R.—Extracti Belladonnæ, . . . . | gr. viij. |
| Ipecac pulv, . . . . . . | gr. x. |
| Zinci Sulphatis, . . . . . | dr. ss.—M. |

Ft. pil. No. 30.

S.—One every hour. HORACE GREEN.

HYPERÆSTHESIA OF STOMACH.—Dr. C. Handfield Jones says:

In the hyperæsthesia of pregnancy, the influence of reflex irritation is evident. Nitrate of silver is a really valuable remedy in cases where pain is distressing; and, as it need only be given for a short time, you run no risk of spoiling your patient's complexion. It may be seconded, however, or replaced, very frequently, by subcutaneous opiates. I have employed them myself with the best effects. Dr. Clifford Allbut writes strongly in their praise. One of his cases was that of a merchant who regarded himself as a hopeless dyspeptic, dreaded his meals, and especially his nights. He was cured (at least for the time) by one-fifth of a grain of morphia subcutaneously at 3 P. M. and 10½ P. M., followed by rest, during ten days, with one-fourth of a grain at night only for ten days more. I prefer myself to make these injections at the epigastrium, but this is not necessary. When the hyperæsthesia expresses itself in immediate vomiting without severe pain, I have great faith in strychnia, administered in doses of one-twentieth of a grain in not more than half an ounce of water. Let me cite the following instance: A slight-made female, aged twenty-two, married four months, subject to indigestion, and weakened by a miscarriage, was brought to me with severe sickness of ten days' duration, no food having been retained the last three. I gave her, in my room, one-twentieth of a grain of strychnia, and made her lie down. After she had retched a few times ineffectually, she became much better, "did not feel at all sick," and took successively four teaspoonfuls of milk before she left. I saw her again in ten days, when she was greatly better, had only been sick once, and was able to take chicken.

LAXATIVE PILL.—

| | |
|---|---|
| R.—Ext. aloes pulv., . . . | oz. ss. |
| Gambogiæ, . . . . | dr. j. |
| Rhei pulv. . . . . | dr. ss. |
| Olei cinnamon, . . . . | gtt. xx. |

Make 120 pills.

The above is the favorite laxative pill of a distinguished lecturer and practitioner.

LAXATIVE MIXTURE.—Prof. Lindsly says the best remedy he has ever tried in habitual constipation, is to take a half drachm of epsom salts, dissolved in half a pint of water—adding ten drops of Elixer of Vitriol—*one hour before breakfast.* The may be smaller the dose the better, provided it will operate. It may be taken for weeks till a cure is effected.

ERGOTIN USED HYPODERMICALLY IN HÆMOPTYSIS.—Dr. C. C. Ritchie, Manchester, England, in speaking of the hypodermic use of ergotin in hæmoptysis, says:

I have adopted it in eight cases with success; a solution of five grains of ergotin in ten minims of distilled water was employed, except in one case, in which unusual irritation was produced by it; I therefore substituted a solution (prepared according to Langenbeck's formula) of three grains of ergotin, in equal parts of glycerine and rectified spirit, which produced very little irritation.

CHILBLAINS.—M. W. E. Schaller says that the fluid concentrated chloride of iron is an unfailing remedy for chilblains, its application to them for a single day effecting a cure. It may also be used with advantage for frostbites.—*Wiener Medizinische Wochenschrift.*

Hyperæsthesia of the Testis, Dr. C. H. Jones says, is a very distressing neurosis; it reacts very injuriously upon the mind, and has in several instances induced sufferers to insist on the removal of the affected organ, which in three cases was found healthy by Sir A Cooper. He considers it of centric origin; but it may be of reflex peripheral origin, as in the following case. A. C. aged 23, was admitted Nov. 13th, 1870. He was in good health until five days previously, when he was suddenly taken with pain in the left testis; it was severe, and the testes began to swell. In a day or two the right became painful also. These symptoms were succeeded by pain in the loins and head. The pain now affected him only when he stood up, or when the testes were pressed. There was very little swelling of either testis. He had some tenderness of the hypogastric region, and a good deal of straining at stool. He had no sleep at night. Temperature 103.5 deg.; pulse 80. He had thirst and anorexia. On the 15th he experienced a kind of tearing sensation at both external abdominal rings, going down into the testes. On the 16th the temperature was 97.2 deg.; pulse 60, weak. The bowels had not been open since admission. His extremities became very cold the previous night; and he then became very hot and burning, and afterwards perspired profusely. He could not stand without support; the room and everything appeared to turn round with him. His testes were small, soft, very tender when touched, and the pain shot up along the cords. The bowels were now well opened with castor oil, and afterwards an opium suppository of one grain was introduced. The testes ceased to be painful about four hours after the bowels acted, before he had the suppository. On the 17th day they bore handling well; he was also less giddy, could walk, but not very steadily. He had no pain anywhere. He was now taking tincture of cinchona and carbonate of iron, and by the 23d was getting quite well. The hyperæsthsia and pain of the testes in this instance were mainly dependent on the irritation caused by feces in the lower bowel; but the action of this cause was probably much promoted by the existence of influenza enfeebling the nervous system.

For Chroic Bronchitis.—

| | |
|---|---|
| R.—Decoc. Polygalæ, . . . | oz. v. |
| Potass. Iodid., . . . . | dr. iij. |
| Tr. opii camphor, . . . | f. oz j. |
| Syrupi Tolutan, . . . | f. oz ij.—M. |

S. Teaspoonful twice a day.

This is highly recommended.

Hyperæsthesia of the Heart.—Dr. C. H. Jones says:

A belladonna plaster is a remedy that generally does good service, and is to be preferred, I think, in most cases, to the subcutaneous opiate, which, in two cases where I tried it, produced more syncope than was at all agreeable to witness. Dr. Clifford Allbutt, however, assures us that subcutaneous injection of morphia in small doses, is very valuable in so called irritable heart, whether this be due to weakness of the organ or instability of its nerves. The first dose administered in this way should not exceed one-tenth of a grain.

Hyperæsthesia of the Rectum.—Of this condition Dr. C. H. Jones remarks:

A sort of diarrhœa which I have occasionally met with, in elderly persons, seems to me attributable with much probability to hyperæsthesia of the *mucous lining of the rectum.* The feces appear natural enough in all respects; but the lower bowel is intolerant of their presence, and, in a fidgety, petulant manner, insists on expelling them much more frequently than is at all convenient. Belladonna has appeared to me of some service in this condition of things, and still more, perhaps, cold sponging of the anus directly after defecation.

Glandular Swellings.—Few remedies will be found more efficacious for promoting the absorption of glandular swellings in the neck, or those of other parts of the body, than the following:

R.—Iodini, . . . . . gr x.

Potass. Iodid., . . . . dr. j.

Liquor potass., . . . . f. oz j.

Syr. Sarsa., . . . . f. oz iij.—M.

S. Tablespoonful twice a day.—Horace Green.

Urticaria.—The following is the treatment of uticaria recommended by the *Journal des Connassances Medicales:* Alkaline baths of bicarbonate of soda (300 to 500 grammes), lotions of vinegar and water (one part of vinegar to three of water), to be applied to the affected region with a sponge when the itching and tingling are excessive. Prurigo, as well as uticaria, yields usually to three or four baths made of 20 grains of corrosive sublimate dissolved in 100 grammes of alcohol at 90 deg. C., to which 300 grammes of distilled water is added. This mixture is put into water extraordinarily used for a bath. Acidulated drinks should be used during the treatment. Meats and vegetables containing nitrogen, such as cabbage, should be avoided.—*The Doctor, Nov.* 1.

Typhoid Fever Treated with Strychnia.—Dr. John E. Owen (Medical Record August 15) says "that during the last four years, both in hospital and private practice, milk and the acid and strychnia mixture have been administered to patients with typhoid fever with success. The mixture is prepared as follows:

R.—Acid. sulph. arom., . . . dr iij.

Strychniæ sulph., . . . gr $\frac{1}{73}$

Syrup. simpli., . . . . oz v.—M.

Dose, a tablespoonful.

There is one noticeable feature in cases treated by strychnia—viz., the dry, brown tongue soon becomes moist, and remains so during the treatment; this is effected, he believes, mainly through the agency of strychnia, by increasing the nutritive and assimilative functions of the system.

New Operation for the Cure of Variocse Veins.—Mr. Stokes has been recently treating varicose veins on a plan which was suggested to him by Sir Dominic Corrigan. It occurred to Sir Dominic that as hemorrhoidal tumors are, as a rule, so successfully treated by the application of strong nitric acid, the application of this acid to varicose veins in other situations would probably be attended with equally good results. In a case which is still under observation in the Richmond Hospital, this plan of treatment has been attended with the happiest results. The patient is a young man, aged 21, and was admitted into the hospital on the 15th of last month. He had a varicose tumor, of the size of a small orange, on the inner aspect of the middle third of the right leg. It had existed for seven years. He suffered also from a large varicose ulcer, which existed over the inner ankle of the same leg; and there was also a second tumor, formed of a cluster of varicose veins, in the right groin. Mr. Stokes performed the operation in the following way. Pressure having been made above and below the tumor, the integuments were raised from the tumor, and an incision, by transfixion, was made over the veins. The *fuming* nitric acid was then applied to the external coats of the veins. No pain attended this application. On the following day the contents of the tumor appeared solidified at the base; and the acid was again applied. The process of solidification then went on rapidly, the tumor at the same time decreasing in size. A week after the operation, some coagulated blood appeared at the site of the operation: and the following day a portion of the vein came away. This was followed by a slight local inflammation; which, however, after a few days, quite subsided. The wound was then for some days dressed with tinct. benzoin co. and glycerine; and it rapidly healed, as did also the ulcer; and the large varicose tumor in the groin entirely disappeared.—*British Medical Journal*, May 28, 1871, p. 550.—*Braithwaite's Retrospect.*

Some of the Ill Effects of Bromide of Potassium.—"Under this heading Mr. T. O. Wood, Medical Superintendent of Dunston Lodge Asylum, publishes a paper, with cases, in the British Medical Journal of October 14, 1871, in which *inter alia*, he says its most dangerous effect is when, after a course of comparatively small doses, which do not seem to be taking any great hold of the system generally, or upon the mental symptoms, to control which it is given, it suddenly, and without apparent cause or warning, displays its cumulative effects, and rapidly reduces the patient to a condition of great bodily prostration, and completely alters the character of the mental symptoms. The physical prostration is at once evident. There is great muscular debility; dimness of sight with dilated pupils; irregular gait, the patient reeling as though intoxicated; whilst nausea, vomiting or purging, with abdominal pain of a low aching character, may also be present, the breath having a peculiar disagreeable odor. Its effects upon the mental symptoms are no less marked. The patient, who was excited, glorying in his imaginary powers of body and mind, becomes despondent, sullen, melancholy, and frequently lachrymose, often even despairing.

In a previous number of the same Journal are cases reported by Witlow Bovis, L.R.C.P., and Dr. R. W. Soss, bearing out these statements except in regard to the suddenness of the symptoms."—*New Remedies.*

For Hysteria.—

R.—Tinct. castori.,

Tinct. assafœtidæ, . . aa f. oz iss.

Aquæ camphoræ, . . . f. oz j.

Spirit ammo. aromat. . . f. oz ss.

Syrupi acaciæ, . . . f. oz iss.—M.

S. Tablespoonful pro re nata.

This is recommended as a "powerful anti-spasmodic."

A Plan for Facilitating the Reduction of Strangulated Hernia by Taxis.—Dr. P. Smyly, in the British Medical Journal for December 25, 1871, reports a plan by which he has successfully reduced strangulated hernia when all other plans had failed. "In inguinal hernia in the male the index finger is applied to the lowest part of the scrotum. This invaginated, the finger being passed behind the testicle and cord up to the external ring. The hernial tumor is then pressed downwards over the finger, toward the back of the hand, so as to make the strictures in the ring tense, and consequently smaller. The invaginated finger is then forced firmly upwards and outwards in the direction of the internal ring. As soon as the finger is firmly grasped the hand should be slightly turned, and the finger pushed toward the middle line. Considerable force may be safely applied in this way, as all the delicate structures are behind the finger, which acts mainly on the stricture. On withdrawing the finger the hernia can usually be easily returned. The same principle is equally applicable to femoral hernia." The advantages claimed for this plan are: 1st. The strangulating portion of the ring is dilated before any pressure is applied to the bowel. 2d. Much greater force may be applied to dilate than could safely be brought to bear when the intestine is employed for dilatation, as in ordinary taxis. 3d. There is much greater probability of returning the bowel into the abdomen in a good condition, and consequently in a number of cases avoiding a dangerous surgical operation.—*Detroit Review of Med.*

Liniment for Rheumatism and Neuralgia.—

| | | |
|---|---|---|
| R.—Olei origani, | | |
| Ticnt. camphoræ, | - - | aa f. oz ss. |
| Granville's Lotion, | - - | f. dr iij. |
| Chloroformi, | - - - | dr iijss. |
| Tinct. aconite, | | |
| Tinct. capsici, | - - | aa f. oz ss. |
| Ol. sassafras, | - - - | dr ss. |
| Lin. safon. comp'd, | - - - | oz j.—M. |

—Prof. Cox.

Arsenic in Multiple Glands.—(*Wien. Med. Woch.*, '71, 14.) Billroth reports the case of a woman, aged forty, who had been in good health during her youth. The disease began ten months before her reception into the clinic, with enlargement of the glands of the neck, ; those of the axilla and groin soon followed. When the patient was recieved into the clinic, the tumors in the neck on both sides had reached the size of the fist, those of the axilla and groin formed swellings of the size of an apple, those at the elbow the size of a hen's egg; and the mesenteric glands seemed enlarged also. The spleen was double the normal size. Examination of the blood showed that no leucæmia was present.

An experiment was first made with quinine, commencing with twelve grammes, gradually increasing to twenty grammes, daily. As this treatment produced no effect, Billroth decided to employ arsenic, which so frequently causes the reabsorption of splenic enlargement in intermittent fever. A mixture of equal parts of Fowler's solution and water was given in doses of five drops, at first, gradually increasing, in the course of four weeks, to twenty drops, and then again returning gradually to the original dose. Two weeks after the commencement of this treatment there was already a marked diminution of all the tumors ; and, when, after two months, the patient was dismissed, there remained only a single gland, of the size of a hazel-nut, all the others having been entirely reduced.

HYPERÆSTHESIA OF THE JOINTS, says Dr. C. H. Jones, is well known to surgeons since the publication of Sir B. Brodie's work on local hysterical affections, which I advise you all to read. The most constant symptom is morbid sensibility of the joints and adjacent parts, which is associated sometimes with more or less general tumefaction, apparently from a turgid state of the small vessels. I have known a condition of this kind, attend d with sanguineous vomiting, yield to carbonate of iron, after an offer of amputation had been (fortunately) declined. I hope none of you will ever make such a *faux pas.*

DR. JOHN WARE'S FORMULA FOR CHLOROSIS.—

| | | |
|---|---|---|
| R.—Ferri citrat., - - - - | | dr ij. |
| Syrupi aurantii, | | |
| Aquæ menth. pip., - - | aa | f. oz ij. |
| Aquæ puræ, - - - | | f. oz iv.—M. |

S. Teaspoonful twice a day.

SULPHITE OF SODA AS A PROPHYLACTIC IN SMALL POX.—Dr. D. P. Boyer (Medical & Surgical Reporter Feb. 17, 1872) writes :

In my article on the treatment of small pox by carbolic acid and sulphite soda, I forgot to mention that I also used sulphite soda as a prophylactic, and in no instance did I have a second case in the family where it was so used—no matter how severe the case under treatment was. My custom is to give from five to twenty grains of the salt, morning and evening, to every member of the family, according to age. It mattered not what the exposure to the contagion was, in no instance was another member of the family attacked.

In one case where the husband had varioloid the wife was obliged to sleep in bed with the patient or on the floor. I advised the former ; gave the sulphite ; notwithstanding she was not vaccinated since a child, and was very much alarmed. She had no symptoms of small pox.

TREATMENT OF FACIAL AND DENTAL NEURALGIA. — This method consists in turning into the meatus auditorious from four to ten drops (according to the age and sensibility of the patient) of the following fluid; then to close the opening of the ear by means of a little cotton, and to cause the patient to hold the head inclined for some minutes to the side opposite to the seat of the pain, so that the liquid may remain in the bottom of the ear. This preparation is thus made:

R.—Ext. opii
Ext. belladonna,
Ext. Stramonii, - - - aa parts j.
Aq. pruni virg., - - - parts xij.
Solve et cola.

Although this preparation may be only extemporaneous, it may nevertheless be preserved, if care is taken to keep it cool, by pouring on its surface from two to four drops of sweet almond oil.

It is very rare, with the use of this liquid, that relief is not obtained in a few minutes, and the patient asleep in half an hour, whatever may have been the severity of the pains, and that without having been in the least danger. Absorption takes place almost as rapidly as from a denuded surface, and it is, therefore, unnecessary to blister the patient when we wish to use narcotics, since they act almost as rapidly by the auditory passage.

If it should happen that, at the end of eight or ten minutes, the pain does not yield to the remedy (which sometimes happens when the quantity used has been too small, or when we have to treat a neuralgia which has already required the use of narcotics in any way), it is necessary to use a second dose, at least equal to the first, but in the opposite case, in order to obtain, promptly, that relief which is only too frequently, momentary, of facial neuralgias of long standing.—*American Practitioner.*

NAUSEA IN PREGNANCY.—(British Medical Journal, March 11.) Mr. T. E. Clark, Surgeon to the Bristol Royal Infirmary, says he has found two grains of extract of white poppy, every four hours, exceedingly successful; also, a pill consisting of creasote, half a minim; bismuth, two grains; extract of hyoscyamus, two grains; every four hours. The patient should be made to lie down for some time after each meal.—*Half yearly Abstr.* July, 1871.

COLLIGUATIVE SWEATS.—M. J. Guyot has found the phosphate of lime to be almost a specific in the night sweats of consumptives, tending at the same time to improve their general health. The dose is from 30 to 90 grains per day.—*Union Med.*

Is Phthisis Contagious?—In reply to this question, Dr. C. W. Stephens, of Charlestown, writes:

I was present at the experiments on inoculation of tubercle by Prof. Villemin, of Paris, in 1865–1868. The inoculated rabbits invariably showed, after a variable time, fine neoplasms of tubercle at the spot of inoculation and in the regionary lymphatic glands, and were either killed or died of phthisis. These experiments have rightly raised the question as to the contagiousness of tuberculosis. I have lately observed a case which seems to favor the affirmative.

Mr. R., aged 29, died of phthisis after three years' sickness. The last few months of his life he had a tuberculous diarrhœa. His expectoration was purulent and profuse. He had returned home from abroad only six months before his death. He was nursed chiefly by his young wife, and assisted by his wife's mother. His wife had a fine constitution, a well-developed body, and had always been healthy. Her mother was likewise a very robust woman of middle age, with no hereditary consumptive tendencies. This young wife was incessant in her devotion to her husband—constantly sitting beside him, breathing his breath, bathing his body, and emptying his sputa and excretions. Near the end of his life she was taken with a slight diarrhœa. After his decease she appeared to go into a decline, and died of acute tuberculosis in about six months. About a year afterwards her mother likewise died of consumption.

It can hardly be said that these two victims of womanly courage and devotion went into a tuberculous decline from the fatigue and exhaustion of self-denying vigils; for nurses in long-continued typhoid fevers and other chronic diseases demanding great fatigue, do not become tuberculous, but they often fall into a typhoid fever. I would be glad to have my professional brethren relate any experience bearing on this point.

For Scarlet Fever.—

| | | |
|---|---|---|
| R.— | Ammon. carb., - - - | scr iiss. |
| | Tinct. opii camph., - - - | f. dr ij. |
| | Vini ipecac., - - - - | f. dr ss. |
| | Aquæ puræ, - - - | f. oz vj.—M. |

S. Tablespoonful every 4 or 6 hours.

It should be taken thus: To one spoonful of the mixture add two ounces of water, sweeten, and mix with it one drachm of lemon juice, and take while effervescing. This is spoken of very highly by an excellent physician of Massachusetts.

"JACKSON'S COUGH SYRUP."—The formula for this preparation has not been uniform, and therefore the Cincinnati College of Pharmacy has recently presented the Academy of Medicine the following formula as an uniform standard, indorsed by the secretary, Mr. James M. Ayers:

| | | |
|---|---|---|
| R.—Fluid extract ipecac, | - - | dr ss. |
| Fluid extract Senegæ (oz i Rad Sen. to f oz i) | | dr iii. |
| Fluid extract Rhei, | - - - | dr iv. |
| Syr. simplex, | - - - | oz xxxi. |
| Morphia Murias, | - - - | gr viii. |
| Ol. sassafras, | - - - | gttæ xxxi. |
| M. ft. Mistura. | | |

And hereafter all prescriptions for this mixture will be prepared on the above formula. There are other preparations of a standard character which should receive the attention of the College of Pharmacy, and we trust they will at an early date.

COLIC IN BABIES.—(*Alexander W. Acheson, M. D.*)—" An ounce of prevention is worth a pound of cure.

The "pound of cure" I leave for another occasion, contenting myself with a few observations on the "ounce of prevention."

If taken in due time, no trouble is more easily controlled than colic. If permitted to exist, not only the child suffers, but the mother, through anxiety and loss of sleep, may be broken down. My attention was first called to colic by my own child, who cried regularly every day, for the first three months of its existence. For it I could get no remedy to cure. Palliatives were numerous. In this case, I observed that as soon as the child was born, its grandmother took it away and fed it. Was that correct?

I had noticed that the calf, the colt, the young of all animals, except man, get no food until nature provides. The first chicken of a brood may be hatched on the seventeenth day, and remain on the nest with the old hen, until the twenty-first without food, and at the end of that time be the strongest, brightest, liveliest chicken in the brood.

A mother's milk may not appear until the third day. Is it proper to let the child go without until the supply comes?

Theoretically, yes; because if Nature intended the child to have food sooner, it would provide it.

But what will be the effect on the child?

This question I determined to answer by an experiment. When my next child was born, I gave explicit directions that it should not have anything to eat except what the mother could supply. It was a difficult matter to surmount the objections of the mother, nurse, friends and neighbors. "The child could not live," was the common expression. But it did live, kept quiet, did not seem to suffer for want of food, and on the third day had its patience rewarded by a full meal.

This child cried during its childhood, from colic, only three times, and in every instance we were able to trace it directly to indigestible food.

I was satisfied; but as "one goose does not bring Spring," so one case does not establish a rule.

In the second case in which I pursued this course, a like result followed. The child was quiet, and the mother happy.

In the third case in which I laid down the law, an old maid got the child, and on the occasion of my next visit I found her feeding it.

"What are you doing with that child?"

"Feeding it."

"What are you giving it?"

"Molasses and water."

"If you should take that dose yourself, what effect would it have?"

"Give me a headache."

"And yet you'll give to that weak, new-born stomach, truck your own will not bear. Did I not tell you not to give it anything?"

"Yes; but you're too young to teach me anything about children."

In this case colic was present to such an extent that the child did little else but cry for four months.

With these few cases I established the rule, never to feed a child anything until the mother's milk comes, and I have not yet encountered the case wherein I had to regret so doing.

The child may be put to the breast immediately, particularly if there is any disposition toward flaccidity of the womb. There will be some material found in the breasts, but not much for two or three days. There is no fear of starvation inside of four days. Instead of growing weak, it will be seen to steadily improve in strength; and another point of importance in following this rule, is that the child never forgets to suck, as it is apt to do if fed with a spoon.

In every case of labor I now attend, I tell the mother frankly, that if she wants her child free from colic she must not feed it, and if she does, she will suffer for it as certainly as fate.

There is a common impression that whatever the mother eats which is liable to produce colic in her, will, through the milk, have the same effect on the child.

If you disable a child's stomach by improper food at the beginning, it is not surprising if anything would on certain occasions give rise to colic. But if a good start is made, and the stomach not injured at birth by "brandy and water," "sugar and water," or "molasses and water," &c., the mother can eat whatever she wishes, and without fear of her child suffering for it.

SODA MINT.—The very popular "soda mint," so much employed as an antacid and carminative for over-fed infants and dyspeptics, was originally a favorite prescription of Dr. George Norris, of Philadelphia. His formula was the following:

R.—Sodæ bicarb., - - - oz ss.
Aquæ menthæ piperi, - - - Oj.—M.

Dose: from a desert-spoonful to a table-spoonful for adults; from half to one teaspoonful for infants.—*Journal of Pharmacy.*

SULPHATE OF IRON IN SUPPURATION.—A child, burned all over, was lately brought into the hospital. The suppuration from the wounds was so profuse and offensive, that the ward, in which he lay, was almost uninhabitable. He was placed in a bath containing two handfuls of sulphate of iron. The cessation of pain was almost immediate; after repeating the bath twice a day, for fifteen or twenty minutes at a time, the suppuration moderated, the fetid odor disappeared, and the patient rapidly recovered.—*Boston Journal of Chemistry.*

ARSENIC IN DYSPEPSIA.—Dr. J. C. Thorowgood, in the *Practitioner*, speaks highly of the action of arsenic in many diseases of the stomach. He has found that one drop doses of Fowler's solution in half an once of infusion of Columba had the effect, in a case he treated, of allaying the pain, stopping the vomiting of food, and enabling the patient to eat and digest small quantities of mutton. He states that the usual irritable tongue, with projecting papillæ and yellow or gray fur, indicate arsenic. The more purely local the gastric symptoms, the better is the chance of arsenic doing good. Where there is much general exhaustion of the system, with disordered urine or hepatic congestion, it does not promise much.

SULPHUROUS ACID IN SYPHILITIC ULCERATION.—Dr. Murchison has recently tested this remedy at the Middlesex Hospital, with a favorable result, in the case of an old woman, with tertiary syphilitic ulceration of the pharynx, involving deeply the posterior pillars of the fauces. Iodide of potassium with chlorate of potash was employed internally, and the sulphurous acid (one part to four of water) was used locally in the form of spray. The ulceration was speedily checked, and continued to improve so long as the application was kept up. When the treatment was omitted the ulceration recommenced.—*Richmond & Louisville Med. Jour.*

An Astringent in Gleet.—Dr. R. S. Johnson extols, for cure of gleet, an astringent injection composed of:

| | | |
|---|---|---|
| R.—Tr. opii, | - - - - | dr j. |
| Tr. catechu, | - - - - | dr ss. |
| Mist. acaciæ, | - - - | oz ij. |

To be used twice daily.

He relates one case in which the discharge ceased after the second application, and did not return.—*Medical Press and Circular.*

Nitrite of Amyl in Epilepsy.—Dr. S. Weir Mitchell (Philadelphia Medical Times, April 15, '72) in speaking of a case of epilepsy, says:

Many remedies have been vainly employed, and even the bromides in full doses, fail to do more than lessen the number of attacks; while strychnia, valerianate of quinia, zinc, and other agents have all alike failed to afford relief.

I made many experiments with a view to cutting short the fit by interfering with the precedent local spasm; but neither blisters along the nerve-tracks which are not tender, nor a ligature tightly applied, proved of a use. I then gave the patient a drachm of chloroform in phial, directing him how to inhale it from a handkerchief, but soon found that he was unable to inhale enough of it to serve his purpose. As a last resort, I gave him in a very small phial three or four drops of nitrite of amyl, and showed him how to inhale it by putting the open phial up one nostril while with one finger he closed the other, and then made a few full inspirations. The first attempt failed, because, as he said, the spasm of the left limb made him nervous. On the second occasion he began to breathe it the instant the fingers twirched—having pulled the cork of the phial with his teeth. In a few moments he felt his face flush, the carotids beat violently, his head felt full, and, the spasm ceasing, the attack at once, and for the first time in his experience, was cut short. Four days later he thus cut short another attack; and the experiment has since succeeded in eleven fits, and failed, from too late use ef the nitrite, in two. Moreover, the attacks have lessened in frequency, and now come on only once in ten to twenty days. Not only is there no evil effect from the drug, but his memory has improved, and his nutrition gained considerably. He is again taking bromide of lithium.

I hesitate, as yet, to speculate on the relations between the physiological influence of this interesting agent and the present views of the mechanism of epilepsy; but I do not feel justified in withholding any knowledge, however limited be the foundation on which it rests, when it has proved so distinctly valuable.

In another class of rare cases it may also prove of service. There are certain epilepsies in which the spasms last for hours—one fit following another. In these I commonly employ, with success, injections under the skin of bromide of lithium, using thirty or forty grains in three or four localities; but I have twice checked these attacks at once by inhalations of the nitrite of amyl. In one of them there was a second fit, but no more—which was unusual. In the other, which lasts always several hours, I used the nitrite at the close of an hour, in the third convulsion. Relaxation instantly occurred; the fit passed off, and no other followed. Ether has been frequently employed in this case; but it merely mitigates the attack, and its use has to be kept up for hours.

Treatment by Physostigma.—Dr. N. S. Davis (Chicago Medical Examiner, April 1,) gives notes of treatment in forty cases of Meningetis under his observation since February 1. From this paper we clip the following:

Recollecting that the Calabar bean had been used with apparent success in tetanus, and that I had used it with benefit in several cases of muscular rigidity from irritation of the nervous centres, I made the following prescription:

R.—Tinct Calabar bean, - - - oz j.
Fl. Ext. Ergot, - - - oz jss.—M.

Gave half a teaspoonful every two hours, and omitted all other medicines. At the end of twenty-four hours the patient was found simply more quiet, the pulse a little slower, and the respiration more regular. The treatment was continued without change. At the end of the second day the patient had ceased to complain of pain in her head, had slept several times for half an hour to an hour; the retraction of the head was decidedly less; mind was clear; the mouth moist; and the urine free; but she seemed feeble. More simple nourishment was ordered, and the medicine continued at intervals of three hours. The case continued to steadily improve, and on the fifth day after the commencement of the use of the Calabar bean and ergot, the muscular rigidity had entirely ceased, and the patient was convalescent, though emaciated and feble. The medicine was contined three times a day several days longer, and a combination of extract of malt and the compound syrup of hypophosphites, in addition to nourishment, were given to aid in restoring nutrition and strength. Since that time I have used the Calabar bean, either alone or in combination with ergot, in nearly all the cases that have come under my care, and certainly with more apparent effect in controlling the disease than by any other remedies I have tried.

PHOSPHORUS FOR SLEEPLESSNESS.—Dr. E Cutter (Boston Journal of Chemistry) writes:

Major A. B., aged about 40 years, served three years in the late war. Being very energetic and trustworthy, his labors were arduous and exhaustive. On his return home he resumed the duties of his trade—that of a carpenter. Engaging in building a hot-house, he was exposed to the sun's heat under glass in the summer time. This exposure was followed by symptoms of disturbance of the nervous system, evincing a loss of nerve force. Naturally of a cool temper, he became excitable. The mathematics of his business became very troublesome, and he found it very difficult to cast accounts or make calculations. His steps were rapid and his manner nervous. There was but little excitement of the pulse: appetite fair; bowels regular. Sleeplessness was the most prominent and perplexing symptom.

Chloral hydrate, bromide of potassium, opium, sulphate of morphia, and valerianate of morphia, were successively tried, without avail.

Finally, reasoning that here was a case in which there was a loss of nerve force, and knowing that phosphorus was a most important nerve food, I concluded to put him on its use. He took one-fiftieth of a grain thrice daily in pill form. In a few days he slept as well as ever, and discontinuing the pills, has since enjoyed his natural sleep.

*Post hoc propter hoc* is not always a reliable guide. Still, as I have witnessed the calmant and hypnotic effects of phosphorus in other cases, I am disposed to attribute the result in this case to the agent employed. As phosphorus is an irritant poison, great caution should be observed in its employment. None but the right preparation should ever be used, and then only under the direction and advice of a competent and regularly educated physician.

CARBOLIZED GARGLE FOR DIPTHERIA, TONSILLITIS, ETC.—Carbolic acid, 20 minims; acetic acid, $\frac{1}{2}$ drachm; honey, 2 fluid ounces; tincture of myrrh, 2 fluid drachms; water, 6 fluid ounces. The carbolic and acetic acids to be well shaken together before the other ingredients are added.—CHARLES SEDGWICK.

Glycerine in Putrid Sore Throat.—Dr. J. D. Palmer, in the Journal of Pharmacy, says: "I have found this an invaluable remedy in putrid sore throat, as well as in many other affections. Not long since a case occurred in which its healing properties were fully tested. The patient, a little girl, seven years of age, had been suffering several days before I saw her, and the various remedies employed had made no impression on the disease. As it was with great difficulty and pain she swallowed, and her pulse being very weak and quick, it was important that the remedy employed should possess healing, nourishing, and antiseptic properties; and glycerine possessing these qualities, was administered in teaspoonful doses every six hours. The first dose caused some smarting, the second less, and before giving the third there was obvious improvement. The case was dismissed in three days."

Santonine in Eye-Disease.—D. Dyce Brown, M. A., M. D., Medical officer to the Aberdeen Dispensary; and Alex. Ogston, M. D., Surgeon to the Aberdeen Infirmary, give (British and Foreign Med. Chir. Rev.) "the following results of all the cases indiscriminately in which it was administered:

| | Cases treated. | Cured or improved. | Failures and unknown results. |
|---|---|---|---|
| Nervous deficiency | 35 | 26 | 9 |
| | | Improved. | Failures. |
| Cataract | 4 | 1 | 3 |

"Although it is not possible from these to particularize the precise form of disease in which it answers best, the trial of the drug has left no doubt in our minds that it possesses a great power over many cases of inflammatory and atrophic alterations of the nervous elements of the eye, and is superior, in the treatment of such, to the remedies ordinarily employed. Santonine also seems to posses a very decided effect on hyperæsthesia of the retina, one case in particular improving rapidly under its use, although other medicines had previously failed totally. It does not seem, where defective color-vision accompanying deficient sight is the result of alterations in the nervons elements of the eye, that improvement in one respect is obtainable without an accompanying amelioration in the other symptoms. The dose employed by us in almost every case has been the same, viz., one grain per diem. This dose was administered at bedtime, to avoid the unpleasant yellow vision caused by even so small a quantity.

"In the course of numerous experiments on animals with this drug, for the purpose of ascertaining, if possible, the mode of production of the santonine yellow vision, it became evident that the seat of this action lies in the retina or brain, since the other parts of the eye preserve their normal tints when the coats of the eye are removed to a small extent behind, so as to admit of the vitrous, lens, aqueous, and cornea being observed by transmitted light. No yellow pigmentation of the macula lutea was ever observed.

"In physiological doses, santonine, in the healthy subject, shows a marked action on the eye and brain. Prominently stand the alterations in color-vision, which are now too well known to require statement in detail. Besides this, as proved by the experiments of Dr. Edmund Rose, of Berlin, the retina becomes hyperæmic, both arteries and veins being very markedly enlarged in rabbits poisoned by this drug. Then also distinctly cerebral symptoms are produced, as headache, dizziness, nausea, and vomiting, the latter evidently not arising from disorder of the stomach, but from brain irritation. Alterations in taste and smell are also produced in some cases, while in animals poisoned by the drug, spasm, first of the cerebral and then of the spinal nerves occur. These facts, showing the elective affinity of the drug for the eye and brain, will tend to confirm the impression, deduced from the cases referred to, that santonine will yet take a prominent place in the treatment of certain forms of eye disease."

Hydrate of Chloral in Rigid Os-Uteri in a Case of Miscarriage.—Dr. M. L. Brown, (Boston Medical & Surgical Journal) reports the following:

Mrs. ——, a well-formed and muscular woman, primipara, miscarried at about the fourth month. I was called at 4 o'clock, A. M. The miscarriage had been in progress three days, and the waters had passed away the day before. On examination the vagina was found hot, though somewhat moist, and the os was too small to admit the tip of the fore-finger; it felt hard, and gave a sensation as if bound round with whip-cord. At the end of two hours, the pains coming regularly and increasing in strength, another examination was made, when I found the same condition as at first. At 8:39, A. M., no progress having been made, I gave one-third of a grain of sulph. morphia, as in labor at full term; I have several times seen the best results from giving an opiate when the pains were strong and the os rigid and unyielding. At 11, A. M., there was no change; all the while the pains occurring regularly every five minutes. I then gave two grains of opium, but no effect towards dilating the os, though it materially increased the strength of the pains. At 1:30, A. M., applied the extract of belladonna after the manner of Prof. P. Dubois (a pellet of the extract the size of a pea on the nail of the index finger is carried to and applied up the os uteri). Towards evening, as this had produced no result, several applications of very warm water were made by means of vaginal douches, with the effect to soften and cause the os to dilate so as to admit the finger. After this, no perceptible progress was made, and I thought of incising the os. The pains had become more frequent, powerful, and were agonizing, and the patient complained of great fatigue, a feeling of complete exhaustion, was very thirsty, had severe headache, the tongue was dry, brown and burning, the abdomen was tender on pressure, and the pulse was hard and frequent.

Having some hydrate of chloral at hand, I gave her forty grains in half a glass of sweetened water; very soon after she passed into a quiet and refreshing sleep. Slept an hour and a half, when she awoke, and immediately after it was discovered that the fœtus was in the bed; slight traction was made upon the slender cord and the placenta was drawn from the vagina. The uterus was found firmly contracted; the tongue was moist, and the headache was gone. The patient made a very quick and perfect recovery.

MEAT EXTRACTS.—Dr. P. Muller, in an essay on meat extracts, considers that they are neither directly nor indirectly food, for they do not contain albuminoid matter,, neither do the nitrogenous principles which they contain arrest disassimulation, that is, they do not prevent the waste of the organic matter which composes the body. In small doses, these extracts are useful by the stimulant action of the potassa salts, which promote digestion and circulation; in strong doses—too large quantity at once—these substances may have a very injurious effect. Medical men should bear in mind that, if given alone, these extracts (and the same applies to beef tea) are no nutriment, and only tend to keep the convalescents weak and not only ill fed, but not at all fed.—*Good Health*, April, 1872.

GANGRENE PRODUCED BY DRESSINGS OF CARBOLIC ACID.—Dr. Tillaux reports, in L'Abeille Medicale, December 11, 1871, two cases in which gangrene followed the application of carbolic acid. In one of the cases the acid used was highly concentrated, —there being, in fact, at the bottom of the containing bottle a sediment of the solid acid. The acid in this condition was applied to slight wounds of the second and third fingers. It did not cause much pain, but the patient, noticing that his finger became black, ceased its use. Loss of the middle finger was the result.

